# Differences in predictive and prospective control strategies by batting skill level during an interception task

**DOI:** 10.3389/fpsyg.2025.1671475

**Published:** 2025-10-03

**Authors:** Yu Sun, Dukchan Jang

**Affiliations:** ^1^College of Physical Education and Health, Yancheng Teachers University, Yancheng, China; ^2^Department of Physical Education, Keimyung University, Daegu, Republic of Korea

**Keywords:** interception task, predictive control, prospective control, expertise, visuomotor coordination

## Abstract

**Introduction:**

Successful interception of fast-moving objects requires both predictive control and online processing of visuomotor information. However, it remains unclear how expertise influences the use of late trajectory information when access to the final portion of the trajectory is restricted. This study investigated skill-level differences in visuomotor coordination under conditions limiting access to the latter portions of a moving stimulus trajectory.

**Methods:**

Twenty participants (10 novices, 10 experts) performed a touchscreen-based interception task involving varying stimulus velocities (0.5, 0.67, and 1.0 m/s) and visibility conditions (stimulus-unhidden vs. stimulus-hidden). Participants tracked a horizontally moving target and attempted to intercept it at a designated target area using a stylus. Eye and hand movement data were recorded to assess gaze behavior, motor accuracy, and eye-hand coordination.

**Results:**

Experts exhibited significantly shorter saccadic latencies, longer gaze durations, and lower gaze error compared to novices. They also demonstrated greater hand accuracy and faster response times, with smaller timing and radial errors. Moreover, eye-hand coupling was more efficient in experts, with shorter temporal coupling and tighter spatial coordination—particularly under stimulus-hidden conditions.

**Conclusions:**

These findings suggest that expertise enhances the integration of perceptual and motor processes, supporting more precise and timely responses. Experts also processed limited visual information more effectively and made better use of online feedback, demonstrating that skilled perception-action coupling relies on flexible integration of predictive planning and online feedback.

## 1 Introduction

Successful interception of a moving target requires precise coordination between the gaze and motor systems (Benguigui and Ripoll, 1998; Panchuk and Vickers, 2009). Baseball is considered a sport that challenges human performance due to the speed of the ball and the complex spatial and temporal demands involved (Gray, 2002; Takeuchi and Inomata, 2009). These characteristics make baseball a valuable experimental model for investigating various interception strategies (Gray, 2002). A key motor mechanism in skilled performers is the efficient use of visual information during perception-action coupling (Lebeau et al., 2016; Lobjois et al., 2005; Sáenz-Moncaleano et al., 2018; Vickers, 2007). In fact, the distinctive eye movement patterns displayed by experts in interception tasks have provided important insights into how such actions are controlled and executed (Ceyte et al., 2017; Land and McLeod, 2000; Piras et al., 2010). However, prior research has frequently relied on either simple, highly predictable tasks (Ak and Kocak, 2010; Müller et al., 2017; Ripoll and Latiri, 1997) or simulated tasks that aim to replicate real-world conditions (Higuchi et al., 2018; Kishita et al., 2020a). Our controlled experimental design, by limiting access to late-phase visual information, allows us to isolate the effects of reduced visual information availability.

One such complex scenario is baseball, where a batter can observe the trajectory of the ball for about 0.4–0.5 s from the pitcher's release point to the home plate (Chen et al., 2021; Ripoll and Latiri, 1997). Given the short time to hit the ball, a common coaching saying to batters is, “Keep your eye on the ball” (Vickers, 2007). However, when dealing with fast-moving targets like in baseball, it is impossible to continuously track the entire flight path of the ball using eye movement strategies that maintain central vision alignment with the moving target (Land and McLeod, 2000). In reality, batters anticipate the ball's destination using a predictive eye movement strategy, shifting their gaze ahead of the target. For example, a baseball batter tracks the ball during its initial flight phase immediately after release, then shifts their gaze to the expected point near home plate where the ball is likely to land, employing a predictive saccadic eye movement strategy (Gray, 2002; Mann et al., 2013). Interestingly, skilled batters initiate this predictive eye movement strategy more quickly than less skilled batters (Ceyte et al., 2017; Vicente et al., 2023), which contributes to more stable tracking of the ball's flight path and improved performance. This predictive strategy suggests that skilled batters have learned the trajectory of the ball and key prediction points through repeated practice (Tochikura et al., 2020). In particular, skilled batters adjust their gaze as the ball approaches home plate, timing their swing precisely, which improves both the accuracy and consistency of their hits (Croft et al., 2010; Kishita et al., 2020b).

While numerous studies have demonstrated that skilled batters employ predictive saccades, there remains ongoing debate as to whether visual tracking can be maintained all the way up to the moment of contact. Although cricket and baseball differ in their spatiotemporal dynamics, a study involving elite cricket batters offers important insights into this question. Mann et al. (2013) reported that batters were capable of generating a second predictive saccade not only toward the bounce point of the ball but also toward the anticipated point of bat-ball contact. This finding extends the earlier work of Land and McLeod (2000), which suggested that tracking the ball after it bounced was generally not possible. These results suggest that in fast-paced interception tasks, skilled performers may either engage in two predictive saccades or sustain visual tracking up to the moment of contact. Mann et al. (2013) proposed that this capacity enables batters to monitor the ball's trajectory using peripheral vision and, as a result, fine-tune their swing as late as possible within the limits of the sensorimotor system. Recent literature has framed successful interception not as a strict dichotomy between predictive and reactive control, but rather as a dynamic interplay existing on a continuum (Márquez et al., 2024). This framework suggests that the balance between proactive planning and reactive adjustments shifts based on the predictability and visibility of the target.

Even if early predictive saccades occur due to the high speed of the ball, the absence of online visual processing during the latter flight phase can significantly impair hitting accuracy. That is, successful hitting performance can be assumed to depend not only on initial prediction but also heavily on the ability to process information during the later phase of ball flight (Gray, 2002; Mann et al., 2013). Indeed, elite hitters often succeed in making accurate contact even when unexpected changes occur in the ball's trajectory. These field observations suggest that visual information processed during the latter part of ball flight plays a critical role in refining predictions about the ball's final location and velocity. When the target's direction constantly changes, initial information alone is insufficient, and the performer should rely on continuously updated information. From the perspective of prospective control, sensory information is constantly gathered and used to adjust the timing of the movement (Beek et al., 2003; Montagne, 2005). This emphasizes an ecological approach to understanding interceptive actions, where adjustments continue to be made based on environmental information right up until the movement is executed.

Despite extensive research on interception tasks, most studies have employed experimental environments in which full information about the stimulus trajectory is provided from start to finish. This methodological approach has limited our ability to clearly determine whether predictive saccades in interception tasks are primarily explained by the predictive control perspective, the prospective control perspective, or an interaction between the two. Moreover, interception tasks that involve mid-flight changes, such as the bouncing of a ball in cricket or tennis (Pinder et al., 2011; Sarpeshkar et al., 2017), may differ in their demands on predictive and prospective information processing compared to tasks like baseball, where the ball's trajectory is relatively continuous. In particular, there remains a lack of research that clearly elucidates differences in information processing between experts and novices under these varying task constraints.

Based on these considerations, our study used a coincidence-anticipation task, where participants were required to intercept a moving target at the designated, fixed interception zone. We hypothesize that, when relying on early visual information under temporally constrained interception tasks, expert performers—due to their greater experience with moving stimuli—initiate predictive saccades more efficiently than novices, characterized by shorter latencies and faster targeting of the critical arrival zone. Moreover, experts are expected to exhibit longer fixation durations at the arrival zone compared to novices. If the processing of subsequent trajectory information depends on skill level, experts are expected to show differences in eye movement strategies between the non-occluded and occluded conditions, whereas novices may not exhibit such differences. These variations in eye movement strategies are expected to ultimately affect the timing and accuracy of interception performance.

The purpose of this study was to investigate skill-level differences in information processing by examining how restricting access to later trajectory information influences the coupling between visual and motor systems in temporally demanding interception tasks.

## 2 Methods

### 2.1 Participants

Twenty-four healthy male university students initially participated in this study and were assigned to two groups: 12 novices (*M*_age_ = 21.3, *SD* = 2.4) with no prior baseball experience and 12 elite players (*M*_age_ = 20.1, *SD* = 2.33) from a local university baseball team. The elite players had over 10 years of baseball experience and were right-handed hitters with a batting average of at least 0.280 over two seasons in the KUSF College Baseball U-League in South Korea, representing a high level of performance in the league. They reported practicing regularly at least 5 days per week. All participants had normal or corrected-to-normal vision and confirmed normal color vision.

The sample size for this study was determined based on a priori power analysis from our prior research using a similar experimental paradigm (Sun et al., 2025). While that study employed a within-subjects design, a G^*^Power analysis indicated a minimum required sample size of 12 for a repeated measures design. To account for the between-subjects group factor in our current mixed-design study and to ensure adequate statistical power for detecting group differences, we aimed to obtain a final sample of at least 10 participants per group. We initially recruited 24 participants to account for potential dropout and data exclusion.

During the experiment, two participants (one from each group) withdrew, and two additional participants (one from each group) were excluded due to abnormal eye movement data. As a result, data from 20 participants (10 per group) were included in the final analysis. Written informed consent was obtained from all participants prior to participation. The study was conducted in accordance with the Declaration of Helsinki and approved by the Institutional Review Board of Keimyung University, South Korea (IRB No. 40525-202409-HR-051-04).

### 2.2 Experimental task and apparatus

**Figure 1:**
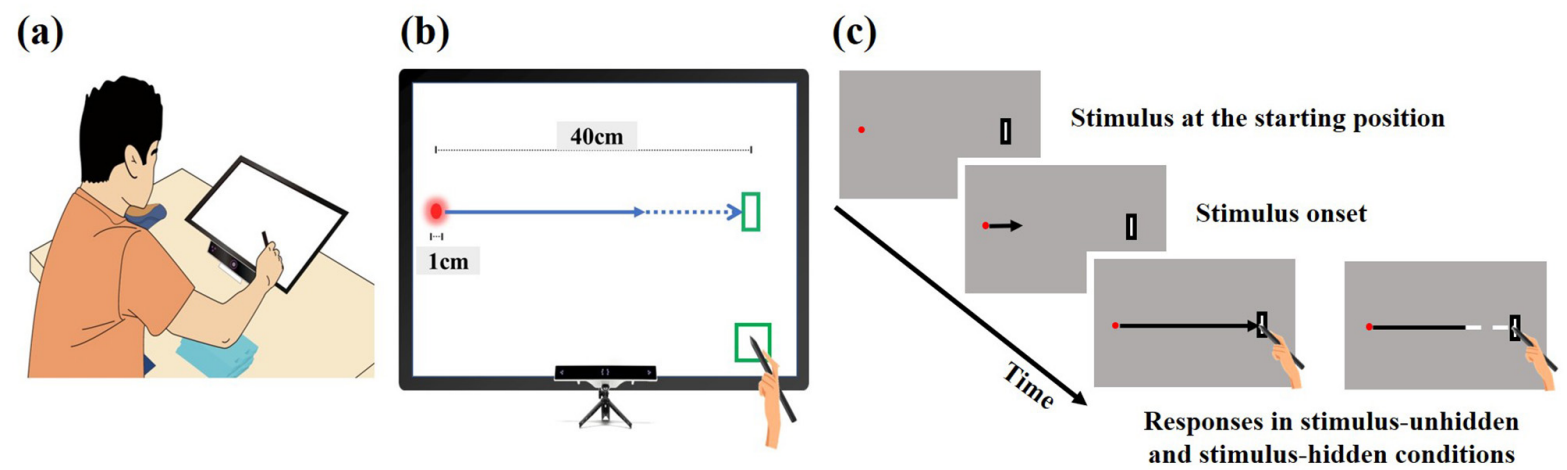
Experimental setup. **(a)** Participants, both novice and expert, were seated in a height-adjustable chair with their chin on a chinrest 30 cm from the touchscreen. **(b)** Eye movements were tracked by an eye-tracker mounted below the touchscreen. The touchscreen presented stimuli moving rightward toward a target area and synchronously measured eye and hand movements. **(c)** In each trial, participants placed a stylus on a preparation area (bottom-right) and shifted their gaze to the stimulus starting position on the left. After 2–3 seconds, the stimulus began moving 40 cm rightward toward a target area at one of three velocities (0.5, 0.67, or 1.0 m/s). In the stimulus-unhidden condition, the stimulus was visible throughout its entire 40 cm trajectory, and participants aimed to hit it upon its arrival at the target area. In the stimulus-hidden condition, the final one-third of the stimulus path was occluded. Participants were still required to hit the stimulus upon its arrival at the predefined target area.

The experimental apparatus, shown in Figure 1, included an eye tracker (GP3 HD, Gazepoint, Inc., Vancouver, Canada) and a touchscreen monitor (U24OLED Edge HDMI, BitM Inc., Korea). A chin rest was used to minimize head movements during responses, and a stylus (CS140B, Wacom Co., Japan) was used by participants to intercept the moving stimulus. The eye tracker's built-in infrared camera detected and tracked the movements of both eyes at a sampling rate of 150 Hz, with an accuracy of 0.5 ° to 1 °. This sampling rate was deemed sufficient for our experimental purpose. While higher sampling rates are necessary for detailed analysis of micro-saccades, a 150 Hz rate is adequate for capturing the latency, duration, and endpoint of larger, visually guided saccades and the continuous dynamics of smooth pursuit, which are the primary gaze behaviors relevant to our interception task.

The touchscreen monitor had a diagonal screen size of 24 inches, a resolution of 1920 x 1080 pixels, and a refresh rate of 60 Hz. The viewing distance from the participant's eye to the screen was maintained at 30 cm by the chin rest, which allowed us to convert pixel coordinates into degree of visual angle (1° = 18.5 pixels).

To examine the spatiotemporal coordination of eye and hand movements during the interceptive task, the eye tracker and touchscreen were synchronized. Both devices were time-synchronized using a shared system clock via a dedicated software script, allowing for precise temporal alignment of eye and hand movement data. The synchronization was confirmed to have a temporal accuracy within ± 6 ms. Data collection for eye and hand movements began when participants contacted the preparation area, located at the bottom-right corner of the touchscreen, with the stylus. At this moment, participants were required to fixate their gaze on the starting position of the stimulus at the left edge of the screen. Recording continued until they completed the response by touching the designated interception target area, located at the top-right corner of the touchscreen. During each trial, a 1 cm diameter stimulus moved horizontally from the left edge of the screen toward the target area on the right, without vertical deviation. From the participant's perspective, this movement covered approximately 67.4 ° of their visual field.

Eye movements were recorded as participants tracked the moving stimulus with their gaze across the screen. Concurrently, hand movements were recorded as participants moved the stylus from the preparation area toward the target area. Eye movement data were processed to identify saccades using an algorithm that applied a velocity threshold of 30 °/s and an acceleration threshold of 8,000 °/s^2^ (Nyström and Holmqvist, 2010). Blinks were identified and removed, and missing data points were handled by linear interpolation. For each response, temporal errors in eye and hand movements were measured in ms. Spatial errors were digitally recorded as x- and y-coordinates in millimeters.

### 2.3 Experimental procedure

Participants were tested individually in a controlled experimental environment. Prior to beginning the task, the researcher provided a verbal explanation and demonstrated the procedure once. Each participant then completed 10 familiarization trials (i.e., hitting a moving target with a stylus on a touchscreen monitor) to ensure proper understanding of the task. During this phase, the researcher monitored participants' performance to confirm comprehension of each condition. Eye tracker calibration was conducted by having participants fixate on nine designated points displayed on the touchscreen monitor. The calibration accuracy was checked every 10 trials, and recalibration was performed only if necessary. Participants were seated on a height-adjustable chair with their chin placed on a chinrest positioned 30 cm from the monitor to minimize head movement.

To further familiarize participants with the experimental setup and procedure, a practice phase comprising 18 trials was conducted. These trials included three repetitions of each combination of stimulus velocity (0.5, 0.67, 1.0 m/s) and stimulus visibility condition (stimulus-unhidden, stimulus-hidden). During this phase, participants were provided with verbal instructions and immediate feedback from the researcher regarding their spatiotemporal response errors to ensure full understanding of the task demands.

Following the practice phase, participants immediately proceeded to the experimental phase, comprising 60 randomized trials. The same stimulus velocity and stimulus visibility conditions used in the practice phase were employed. Trial sequences were randomized using Microsoft Excel and were not disclosed to participants.

In this study, the stimulus moved linearly at a constant speed, with durations ranging from approximately 400 ms (1.0 m/s) to 800 ms (0.5 m/s) over a distance of 40 cm. At the beginning of each trial, the researcher gave a verbal “ready” cue, prompting participants to place the stylus at the preparation area, located at the bottom-right corner of the monitor. Participants were instructed to visually track the moving stimulus as it traveled from the left edge (i.e., starting position) of the monitor toward the target area (3 cm × 9 cm), located at the top-right corner. Simultaneously, they were to move the stylus from the preparation area to the target area, all while keeping their upper body and head stationary. The task required accurately intercepting the moving stimulus with the stylus at the target area. In the stimulus-unhidden condition, the entire 40 cm trajectory of the stimulus was visible. In contrast, in the stimulus-hidden condition, the final one-third of the stimulus path (i.e., the last 13 cm) was occluded, leaving only the first 27 cm visible (see Figure 1).

No feedback regarding spatiotemporal response errors was provided during the experimental phase to avoid influencing participants' control strategies. To maintain motivation and reinforce task goals, participants were verbally reminded every 10 trials: “Your task is to hit the moving stimulus with a stylus precisely when it reaches the target area.” Participants were given 1 min rest breaks after every 20 trials, during which they could rest their arms on the table. The entire experiment lasted approximately 20 mins. After completing the experiment, participants received a brief debriefing.

### 2.4 Data analysis

The collected data were categorized into three domains to investigate skill-level differences in visuomotor coordination during the interception task: (1) eye movement characteristics observed throughout the execution of each trial; (2) the spatial and temporal error and response timing of hand movements; (3) the coupling of eye and hand movements at the point of interception.

#### 2.4.1 Eye movement characteristics

(a) Saccadic latency (ms) was defined as the time elapsed between the stimulus onset and the initiation of a saccadic eye movement. (b) Gaze duration (ms) was defined as the total time the gaze remained fixated within the target area after arriving at the area, calculated as the difference between the time of stylus contact and the time of gaze arrival at the target. (c) Gaze error (mm) represented the spatial discrepancy between the final point of gaze and the actual location where the stimulus arrived at the target area. This error was computed using the following formula:


$$\begin{array}{l} Gaze\;error=\sqrt{x^{2}+\;y^{2}} \end{array}$$

where *x* and *y* represent the horizontal and vertical distances (in mm) between the two respective points.

#### 2.4.2 Spatiotemporal error and response timing

(a) Timing error (ms) was defined as the absolute difference in time between the moment the stimulus reached the target area and the point at which the stylus made contact with the touchscreen. (b) Radial error (mm) represented the spatial discrepancy between the location of stimulus arrival at the target area and the point of stylus contact at the end of the response. This error was calculated using the following formula:


$$\begin{array}{l} Radial\;error=\sqrt{x^{2}+\;y^{2}} \end{array}$$

where *x* and *y* correspond to the horizontal and vertical distances, respectively, between the stimulus endpoint and the stylus contact point. (c) Reaction time (ms) was measured as the interval between stimulus onset and the initiation of stylus movement (i.e., when the stylus was lifted from the preparation area). (d) Movement time (ms) denoted the time taken from lifting the stylus to completing the response at the target area.

#### 2.4.3 Eye-hand coupling

(a) Temporal coupling (ms) was assessed by quantifying the time lag between the eye and hand at the endpoint of the movement. Specifically, it was computed by subtracting the time the gaze reached the target area from the moment the stylus contacted the touchscreen. (b) Spatial coupling (mm) captured the degree of spatial alignment between gaze and hand at response completion. This was calculated as the Euclidean distance between the final gaze position and the stylus contact point, using the following formula:


$$\begin{array}{l} Spatial\;coupling=\sqrt{x^{2}+\;y^{2}} \end{array}$$

where *x* and *y* denote the horizontal and vertical deviations, respectively, between the gaze location and the stylus endpoint.

A three-way mixed-design ANOVA was conducted on each dependent variable, with group (novice, expert) as a between-subjects factor, and stimulus velocity (0.5, 0.67, 1.0 m/s) and stimulus visibility (stimulus-unhidden, stimulus-hidden) as within-subjects factors. To ensure transparency and reproducibility, all ANOVA results are reported with exact degrees of freedom, F-statistics, and effect sizes. A 95% confidence interval for each effect size is also provided. When the assumption of sphericity was violated, the Greenhouse-Geisser correction was applied. Pairwise comparisons for significant main effects and interactions were performed using Bonferroni-adjusted *post hoc* tests to control for the family-wise error rate. Statistical significance was determined using an alpha level of 0.05. All analyses were performed in IBM SPSS Statistics Version 30, and results were reported as means and standard errors.

## 3 Results

### 3.1 Eye movement characteristics

#### 3.1.1 Saccadic latency

Significant main effects were found for group, *F*_(1, 18)_ = 114.63, *p* < 0.001, η_p_^2^ = 0.889, 95% CI [0.713, 0.923], and stimulus velocity, *F*_(2, 36)_ = 25.58, *p* < 0.001, η_p_^2^ = 0.587, 95% CI [0.357, 0.720]. *Post-hoc* tests showed that saccadic latency was significantly shorter in the expert group than in the novice group (*p* < 0.001), and significantly decreased as stimulus velocity increased (*p* < 0.001). No significant main effect of stimulus visibility was found, *F*_(1, 18)_ = 2.03, *p* = 0.171, η_p_^2^ = 0.102, 95% CI [0.000, 0.394].

**Figure 2:**
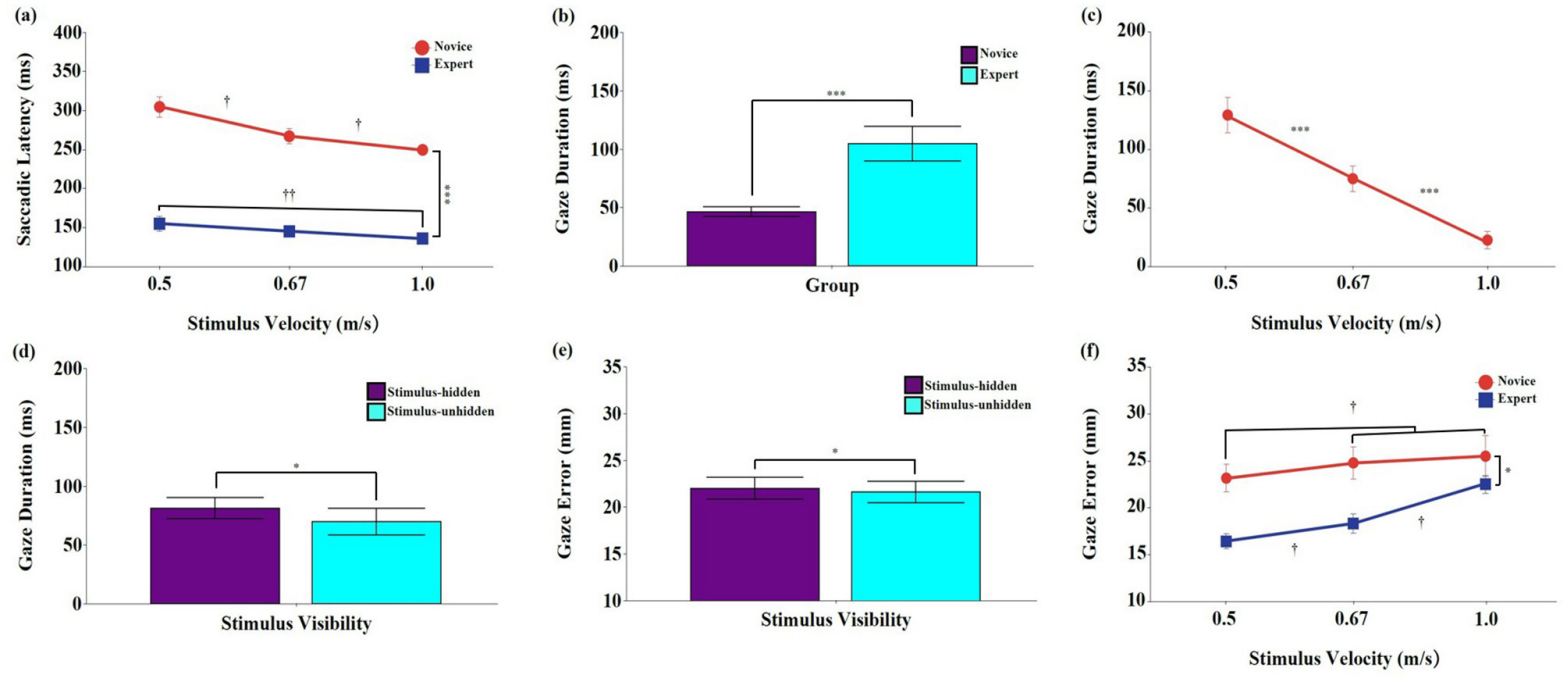
Means and standard errors of eye movement characteristics during the interception task. **(a)** Saccadic latency (ms); **(b–d)** Gaze duration (ms); **(e, f)** Gaze error (mm). Stimulus velocities were 0.5, 0.67, and 1.0 m/s. Error bars represent standard errors. *Novice:* No baseball experience; *Expert:* Members of a local university baseball team. *Stimulus-unhidden:* Stimulus was visible throughout its entire 40 cm trajectory; *Stimulus-hidden:* The final one-third of the stimulus's path was occluded. The asterisks and daggers indicate significant differences. *p < 0.05, ***p < 0.001. ^†^*p* < 0.05, ^††^*p* < 0.01.

A significant interaction between group and stimulus velocity was found, *F*_(2, 36)_ = 6.25, *p* < 0.05, η_p_^2^ = 0.258, 95% CI [0.034, 0.459] (Figure 2a). *Post-hoc* comparisons indicated that the expert group exhibited significantly shorter saccadic latencies than the novice group across all stimulus velocities (*p* < 0.001). Within the novice group, saccadic latency significantly decreased as stimulus velocity increased (*p* < 0.05). In contrast, within the expert group, latency at 1.0 m/s was significantly shorter than at 0.5 m/s (*p* < 0.01); however, no significant differences were observed between 0.5 and 0.67 m/s, or between 0.67 m/s and 1.0 m/s (*p* > 0.05). No significant interactions were found for group × stimulus visibility, stimulus velocity × stimulus visibility, or the three-way interaction (*p* > 0.05).

#### 3.1.2 Gaze duration

The analysis revealed significant main effects of group, *F*_(1, 18)_ = 14.47, *p* < 0.001, η_p_^2^ = 0.446, 95% CI [0.105, 0.673], stimulus velocity, *F*_(2, 36)_ = 56.88, *p* < 0.001, η_p_^2^ = 0.760, 95% CI [0.605, 0.840], and stimulus visibility, *F*_(1, 18)_ = 7.37, *p* < 0.05, η_p_^2^ = 0.291, 95% CI [0.014, 0.565] (Figures 2b–d). *Post-hoc* tests showed that the expert group exhibited significantly longer gaze durations than the novice group (*p* < 0.001). Gaze duration also significantly decreased as stimulus velocity increased (*p* < 0.001), and was significantly longer in the stimulus-hidden condition than in the stimulus-unhidden condition (*p* < 0.05). No significant interaction effects were observed (*p* > 0.05).

#### 3.1.3 Gaze error

Significant main effects were found for group, *F*_(1, 18)_ = 7.32, *p* < 0.05, η_p_^2^ = 0.289, 95% CI [0.013, 0.564], and stimulus velocity, *F*_(2, 36)_ = 66.28, *p* < 0.001, η_p_^2^ = 0.786, 95% CI [0.647, 0.858]. *Post-hoc* tests showed that gaze error was significantly lower in the expert group than in the novice group (*p* < 0.05), and significantly greater at higher stimulus velocities (*p* < 0.001). A significant main effect of stimulus visibility was also found, *F*_(1, 18)_ = 5.85, *p* < 0.05, η_p_^2^ = 0.340, 95% CI [0.000, 0.530] (Figure 2e), and gaze error was significantly greater in the stimulus-hidden condition than in the stimulus-unhidden condition (*p* < 0.05).

A significant interaction between group and stimulus velocity was found, *F*_(2, 36)_ = 16.12, *p* < 0.001, η_p_^2^ = 0.473, 95% CI [0.218, 0.636] (Figure 2f). *Post-hoc* comparisons indicated that the expert group showed significantly lower gaze error than the novice group across all stimulus velocities (*p* < 0.05). Within the novice group, gaze error was significantly lower at 0.5 m/s than at 0.67 m/s and 1.0 m/s (*p* < 0.05). In contrast, in the expert group, gaze error significantly increased as stimulus velocity increased (*p* < 0.05). No significant interactions were found for group × stimulus visibility, stimulus velocity × stimulus visibility, or the three-way interaction (*p* > 0.05).

### 3.2 Spatiotemporal error and response timing

#### 3.2.1 Timing error

**Figure 3:**
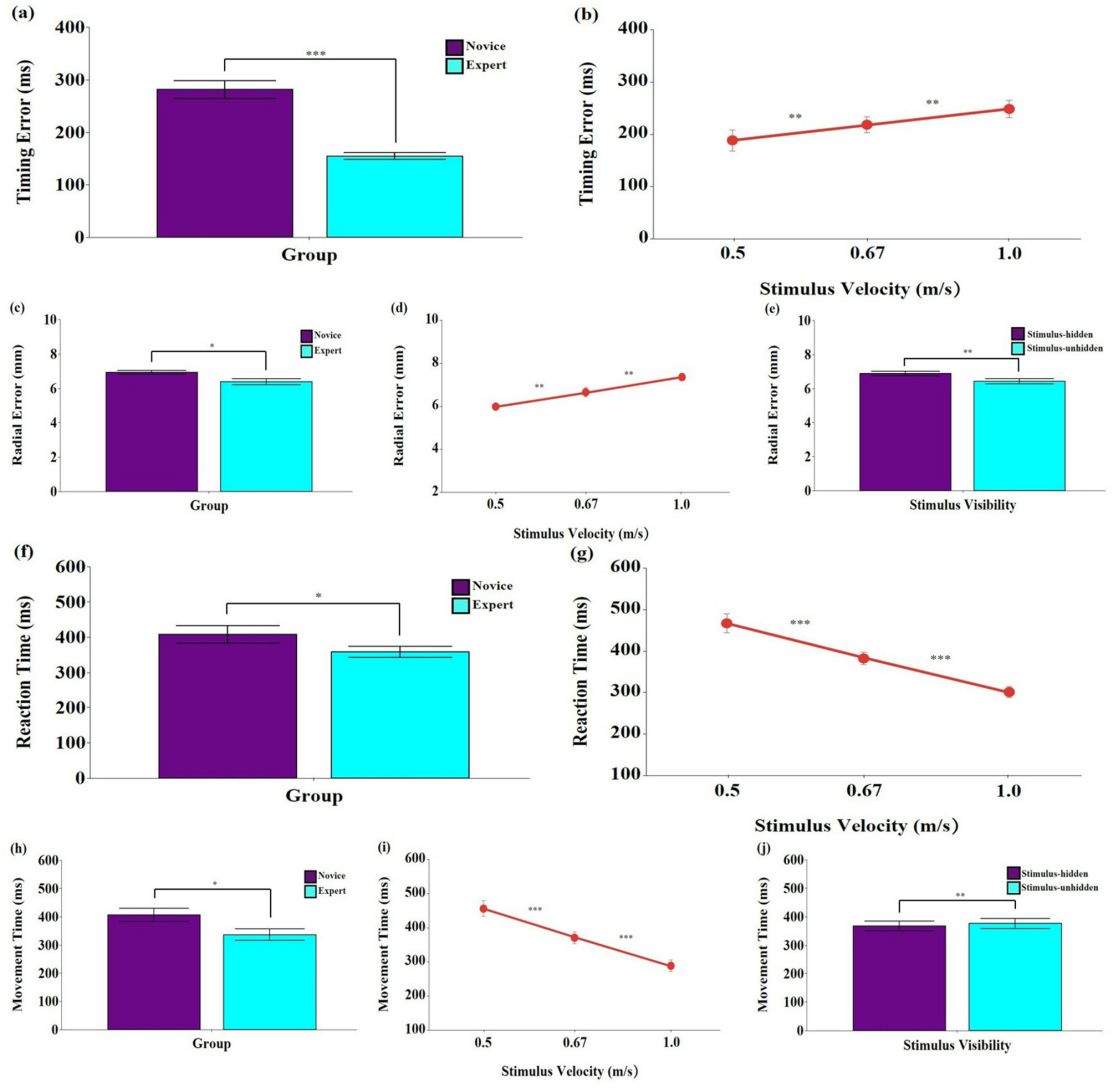
Means and standard errors of spatial and temporal errors and response timing of hand movements during the interception task. **(a, b)** Timing error (ms); **(c–e)** Radial error (mm); **(f, g)** Reaction time (ms); **(h–j)** Movement time (ms). Stimulus velocities were 0.5, 0.67, and 1.0 m/s. Error bars represent standard errors. *Novice:* No baseball experience; *Expert:* Members of a local university baseball team. *Stimulus-unhidden:* Stimulus was visible throughout its entire 40 cm trajectory; *Stimulus-hidden:* The final one-third of the stimulus's path was occluded. The asterisks and daggers indicate significant differences. *p < 0.05, **p < 0.01, ***p < 0.001.

The analysis revealed significant main effects of group, *F*_(1, 18)_ = 49.69, *p* < 0.001, η_p_^2^ = 0.734, 95% CI [0.470, 0.848], and stimulus velocity, *F*_(2, 36)_ = 26.57, *p* < 0.001, η_p_^2^ = 0.596, 95% CI [0.369, 0.727] (Figures 3a, b). *Post-hoc* tests showed that the novice group exhibited significantly greater timing errors than the expert group (*p* < 0.001). Timing error also increased significantly as stimulus velocity increased (*p* < 0.01). No significant main effect of stimulus visibility was found, *F*_(1, 18)_ = 1.37, *p* = 0.257, η_p_^2^ = 0.071, 95% CI [0.000, 0.356]. All interaction effects were non-significant (*p* > 0.05).

#### 3.2.2 Radial error

The analysis revealed significant main effects of group, *F*_(1, 18)_ = 7.31, *p* < 0.05, η_p_^2^ = 0.289, 95% CI [0.013, 0.563], stimulus velocity, *F*_(2, 36)_ = 21.50, *p* < 0.001, η_p_^2^ = 0.544, 95% CI [0.302, 0.689], and stimulus visibility, *F*_(1, 18)_ = 9.19, *p* < 0.01, η_p_^2^ = 0.338, 95% CI [0.034, 0.599] (Figures 3c–e). *Post-hoc* tests showed that the expert group exhibited significantly smaller radial errors than the novice group (*p* < 0.05). Radial error increased significantly with higher stimulus velocities (*p* < 0.01), and was greater in the stimulus-hidden condition than in the stimulus-unhidden condition (*p* < 0.01). No significant interaction effects were found (*p* > 0.05).

#### 3.2.3 Reaction time

The analysis revealed significant main effects of group, *F*_(1, 18)_ = 5.28, *p* < 0.05, η_p_^2^ = 0.227, 95% CI [0.000, 0.507], and stimulus velocity, *F*_(2, 36)_ = 79.97, *p* < 0.001, η_p_^2^ = 0.816, 95% CI [0.700, 0.878] (Figures 3f, g). *Post-hoc* tests showed that the expert group exhibited significantly shorter reaction times than the novice group (*p* < 0.05). Reaction time also decreased significantly as stimulus velocity increased (*p* < 0.001). No significant main effect of stimulus visibility was found, *F*_(1, 18)_ = 4.01, *p* = 0.060, η_p_^2^ = 0.182, 95% CI [0.000, 0.454]. All interaction effects were non-significant (*p* > 0.05).

#### 3.2.4 Movement time

The analysis revealed significant main effects of group, *F*_(1, 18)_ = 5.21, *p* < 0.05, η_p_^2^ = 0.225, 95% CI [0.060, 0.491], stimulus velocity, *F*_(2, 36)_ = 67.88, *p* < 0.001, η_p_^2^ = 0.790, 95% CI [0.640, 0.861], and stimulus visibility, *F*_(1, 18)_ = 7.33, *p* < 0.05, η_p_^2^ = 0.289, 95% CI [0.020, 0.545] (Figures 3h–j). *Post-hoc* tests showed that the expert group exhibited significantly shorter movement times than the novice group (*p* < 0.01). Movement time also decreased significantly with increasing stimulus velocity (*p* < 0.001), and was significantly longer in the stimulus-unhidden condition than in the stimulus-hidden condition (*p* < 0.01). No significant interaction effects were found (*p* > 0.05).

### 3.3 Eye-hand coupling

#### 3.3.1 Temporal coupling

**Figure 4:**
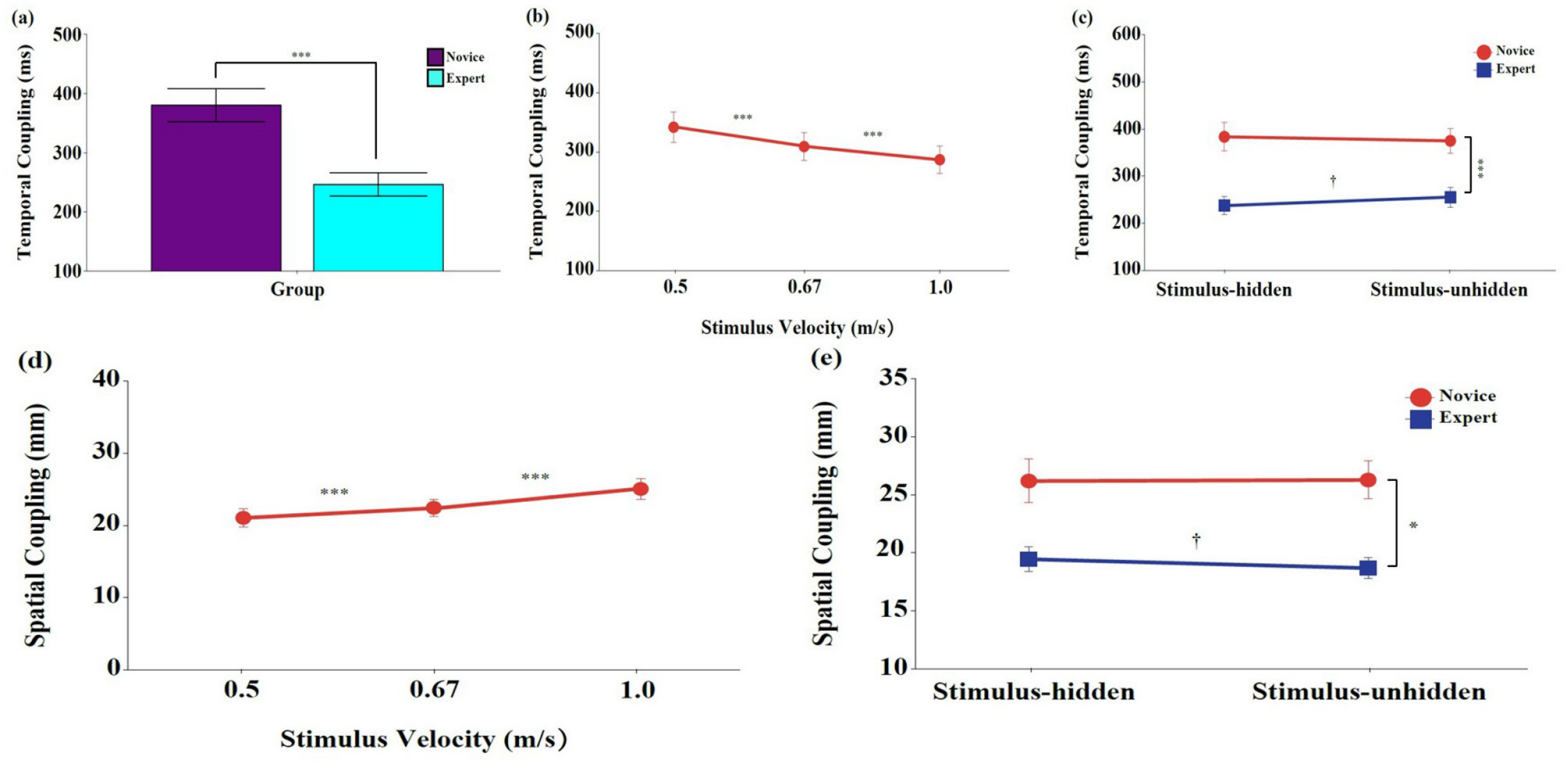
Means and standard errors of eye-hand movement coupling during the interception task. **(a–c)** Temporal coupling (ms); **(d, e)** Spatial coupling (mm). Stimulus velocities were 0.5, 0.67, and 1.0 m/s. Error bars represent standard errors. *Novice:* No baseball experience; *Expert:* Members of a local university baseball team. *Stimulus-unhidden:* Stimulus was visible throughout its entire 40 cm trajectory; *Stimulus-hidden:* The final one-third of the stimulus's path was occluded. The asterisks and daggers indicate significant differences. *p < 0.05, ***p < 0.001. ^†^*p* < 0.05.

Significant main effects were found for group, *F*_(1, 18)_ = 14.99, *p* < 0.001, η_p_^2^ = 0.454, 95% CI [0.133, 0.676], and stimulus velocity, *F*_(2, 36)_ = 7.32, *p* < 0.01, η_p_^2^ = 0.289, 95% CI [0.047, 0.462] (Figures 4a, b). *Post-hoc* tests showed that temporal coupling was significantly longer in the novice group than in the expert group (*p* < 0.001), and significantly decreased as stimulus velocity increased (*p* < 0.001). No significant main effect of stimulus visibility was found, *F*_(1, 18)_ = 0.85, *p* = 0.367, η_p_^2^ = 0.045, 95% CI [0.000, 0.303].

A significant interaction between group and stimulus visibility was observed, *F*_(1, 18)_ = 6.58, *p* < 0.05, η_p_^2^ = 0.268, 95% CI [0.008, 0.543] (Figure 4c). *Post-hoc* comparisons indicated that experts consistently exhibited significantly shorter temporal coupling than novices across both stimulus visibility conditions (*p* < 0.001). In the expert group, temporal coupling was significantly longer in the stimulus-unhidden condition than in the stimulus-hidden condition (*p* < 0.05), whereas no significant difference was found between the two conditions in the novice group (*p* > 0.05). No other interaction effects reached significance (*p* > 0.05).

#### 3.3.2 Spatial coupling

Significant main effects were found for group, *F*_(1, 18)_ = 11.70, *p* < 0.01, η_p_^2^ = 0.394, 95% CI [0.070, 0.632], and stimulus velocity, *F*_(2, 36)_ = 26.75, *p* < 0.001, η_p_^2^ = 0.598, 95% CI [0.372, 0.728] (Figure 4d). *Post-hoc* tests revealed that spatial coupling was significantly lower in the expert group than in the novice group (*p* < 0.01), and significantly higher at faster stimulus velocities (*p* < 0.001). A significant main effect of stimulus visibility was also observed, *F*_(1, 18)_ = 7.17, *p* < 0.05, η_p_^2^ = 0.285, 95% CI [0.012, 0.556], and spatial coupling was significantly greater in the stimulus-hidden condition than in the stimulus-unhidden condition (*p* < 0.05).

A significant interaction between group and stimulus visibility was found, *F*_(1, 18)_ = 8.61, *p* < 0.01, η_p_^2^ = 0.324, 95% CI [0.040, 0.577] (Figure 4e). *Post-hoc* comparisons indicated that the expert group showed significantly lower spatial coupling than the novice group across both stimulus visibility conditions (*p* < 0.05). Within the expert group, spatial coupling was significantly greater in the stimulus-hidden condition than in the stimulus-unhidden condition (*p* < 0.05). In contrast, the novice group showed no significant difference in spatial coupling between the two visibility conditions (*p* > 0.05). No significant interactions were found for group × stimulus velocity, stimulus velocity × stimulus visibility, or the three-way interaction (*p* > 0.05).

## 4 Discussion

Successfully intercepting a moving stimulus requires not only the ability to process visual information rapidly and accurately but also the precise coordination of eye and hand movements (Castiello, 2005; Land and Tatler, 2009). When a stimulus moves along a predictable trajectory at a constant speed, individuals typically rely on a predictive strategy, using initial velocity and trajectory cues to anticipate the stimulus's future location (Bosco et al., 2015). In contrast, when the velocity or trajectory of the stimulus is uncertain, performers are likely to adopt a prospective strategy that involves continuously monitoring the stimulus and adjusting their actions through online control—an approach that demands more complex perception-action integration (Beek et al., 2003; Dessing et al., 2009). However, rather than a strict dichotomy between prediction and reaction, our findings suggest that interception behavior exists along a prediction-feedback continuum (Márquez et al., 2024), with individuals flexibly combining anticipatory positioning and feedback-based adjustments depending on task conditions and their level of expertise. For example, when the latter part of the trajectory was occluded, expert performers were able to combine predictive gaze strategies with effective processing of online information, enabling them to execute spatiotemporal eye—hand coordination more efficiently. In contrast, novices exhibited limitations in predictive eye movements and failed to make effective use of available online information. These findings suggest that an individual's ability to cope with uncertainty in visual information is tied to their skill level (Mann et al., 2007), and that skilled perception-action coupling relies on the flexible integration of predictive planning and online feedback, rather than on one strategy alone.

Experts initiated predictive saccades toward the target area significantly earlier than novices, replicating previous findings that individuals with higher skill levels can anticipate critical task-relevant events more swiftly (Land and McLeod, 2000; Mann et al., 2007). The influence of speed on this strategy was particularly pronounced under high task difficulty (i.e., fast stimulus velocity), where experts demonstrated a significantly earlier onset of predictive saccades compared to novices. While experts' saccadic latencies decreased significantly only at the highest speed (1.0 m/s), their ability to maintain swift responses across a range of velocities suggests a stable visuomotor strategy. This stability stands in contrast to novices, who showed a more pronounced reduction in saccadic latency as stimulus velocity increased, yet their latency remained considerably longer compared to experts. This temporal lag likely contributed to less accurate subsequent performance. Such differences in temporal scaling between groups may be linked to the entrainment of visuomotor responses to target speed (Treviño and Márquez, 2025), suggesting that skilled performers achieve a more stable and efficient coupling between their actions and the dynamic properties of the stimulus. Moreover, earlier initiation of predictive saccades may have enabled more stable and sustained fixations at critical locations, thereby facilitating peripheral monitoring of the object's trajectory—an efficient visuomotor strategy (Bahill and LaRitz, 1984; Mann et al., 2013). Such a strategy likely allowed experts to achieve superior interception performance by integrating perceptual and action processes in a more refined and efficient manner than novices.

Previous studies have shown that the ability to maintain gaze on a target area during object interception is a critical feature distinguishing skilled performers from novices (Mann et al., 2013). Building on our findings that experts initiated predictive saccades earlier than novices, the present study further examined how gaze fixation strategies differed temporally across skill levels at the target area. Specifically, we analyzed the temporal gap between eye arrival and stimulus arrival, as well as the gap between eye arrival and hand movement completion. Results revealed that expert performers exhibited significantly longer eye-stimulus arrival gaps compared to novices (see Figure 2b), indicating that experts fixated on the predicted target location earlier and waited for the stimulus to arrive. Since stimulus arrival time at the target was constant across trials due to the controlled movement speeds (0.5, 0.67, and 1.0 m/s), this longer gap suggests that experts consistently anticipated the stimulus arrival and established steady fixation in advance—a strategy aligned with early predictive saccade initiation (Land and McLeod, 2000; Vickers, 2007). In contrast, analysis of the eye-hand arrival gap showed a different pattern. Experts demonstrated significantly shorter intervals between eye arrival and hand movement completion compared to novices (see Figure 4a). While novices exhibited slower hand responses, leading to a longer eye-hand arrival gap, experts coordinated their hand movements more closely following visual fixation, reflecting faster response execution once fixation was established. This indicates that experts coupled their early predictive visual fixation with more efficient motor planning, demonstrating a key point along the prediction-feedback continuum—a sophisticated transition from anticipatory planning to motor execution (Márquez et al., 2024).

Another key finding was the strategic adaptation observed in skilled performers depending on the availability of visual information. It is important to note that our manipulation reduced visual information, not the fundamental motion predictability of the stimulus. Our findings thus provide insights into how individuals respond to sensory limitations. When the final segment of the stimulus was hidden, skilled participants demonstrated shorter temporal coupling between eye and hand movements, suggesting that they may have shortened the compensation time for their motor responses in the absence of visual information. In contrast, novices showed no notable change in strategy across visibility conditions, indicating a limited ability to adjust their responses under restricted visual input. This lack of adaptability reflects their underdeveloped strategic flexibility. These findings indicate that experts not only rely on predictive visual strategies but also demonstrate the ability to adapt their eye-hand coordination in response to momentary changes in stimulus visibility. Such online modulation of coordination, depending on available visual information, appears to be a crucial aspect of their efficient integration of predictive and online control mechanisms (Márquez et al., 2024).

The temporal strategies observed in expert performers also manifested consistently across spatial dimensions. Analyses of gaze-stimulus and gaze-hand spatial errors provided a more detailed understanding of how spatiotemporal coordination varies by skill level during perception-action coupling. Expert participants initiated saccadic eye movements toward the target area more rapidly based on early stimulus cues, resulting in consistently smaller spatial errors at the moment of interception compared to novices. This finding, aligned with earlier temporal patterns, suggests that expert performance is not merely characterized by faster responses, but by the ability to accurately predict and align gaze and hand movements with the expected stimulus location (Binaee and Diaz, 2019; Mann et al., 2007). Experts also maintained relatively consistent spatial performance across varying task conditions such as changes in stimulus visibility (Stone et al., 2017). These findings highlight the expert's ability to optimize spatiotemporal performance under constraints by leveraging experience-based predictive strategies, ensuring both spatial accuracy and temporal efficiency.

Further analysis of gaze-hand spatial errors revealed a significant interaction between group and stimulus visibility, indicating that experts and novices responded differently to changes in visual availability. Novices showed no significant change in spatial performance across visibility conditions, reflecting a limited capacity to adapt strategically under reduced visual input (Furley and Wood, 2016). In contrast, experts exhibited a slight increase in gaze-hand spatial error under occluded conditions. This pattern appears to reflect a deliberate strategy of utilizing available visual information until the moment of occlusion, then regulating actions based on internally generated sensory predictions (Hodges et al., 2004; Orban de Xivry et al., 2008). In other words, rather than relying solely on reflexive responses, experts integrated predictive sensory cues with fine-tuned control mechanisms, allowing them to sustain effective performance even under visually constrained conditions (Hadjidimitrakis et al., 2019; Klautke et al., 2023).

In addition to the visuomotor behavioral outcomes, skilled participants also demonstrated overall superior performance in actual hand movements compared to novices. Specifically, experts exhibited greater accuracy in both temporal error (i.e., timing error) and spatial error (i.e., radial error) at the target area (see Figures 3a–c). These findings suggest that degraded hitting performance in interception tasks may stem from both spatial and temporal inaccuracies, potentially influenced by the timing of predictive saccade initiation during visuomotor behavior (Land and McLeod, 2000; Mann et al., 2007). Delayed initiation of predictive saccades may not only postpone fixation on the target area or its vicinity but also disrupt early processing of motion stimuli, negatively affecting both spatial and temporal precision (Hayhoe and Ballard, 2005; Sun et al., 2025). The current findings indicate that performance deterioration is not solely tied to the initial trajectory of the stimulus. In fact, while timing error did not significantly differ between the stimulus-hidden and stimulus-unhidden conditions, radial error was greater in the stimulus-hidden condition, where processing of subsequent visual information was restricted. This suggests that the spatial precision of motor execution may depend more heavily on prospective, online feedback mechanisms, whereas temporal accuracy is more reliant on predictive control (Elliott et al., 2001).

Furthermore, reaction time—defined as the interval between stimulus onset and movement initiation—reflects processes of information encoding and decision-making (Botwinick and Thompson, 1966; Luce, 1991). In the present study, reaction time tended to decrease as the speed of the moving stimulus increased, and expert participants showed significantly shorter reaction and movement times than novices. These results suggest that higher stimulus velocity may reduce the time required for cognitive processing, and that skilled individuals, drawing on their extensive experience, can make more rapid decisions and initiate faster motor responses under time-constrained conditions (Abernethy, 1990; Chen et al., 2021; Ericsson et al., 1993).

Taken together, this study demonstrates that perceptual-motor coordination strategies in temporally constrained interception tasks may vary according to the level of expertise. The findings support the concept of a prediction-feedback continuum, where expert performers achieve a more flexible and robust integration of predictive and prospective control. By initiating predictive saccades earlier and maintaining stable fixation, experts can adapt their visuospatial coupling between gaze and hand movements in response to limited visual information, a key distinction from novices. This highlights that skilled performance is not merely about faster reactions, but about a more refined and adaptable strategy for integrating visual information and motor execution.

## 5 Conclusion

The findings of this study underscore the critical role of early trajectory predictability in successful performance during interception tasks. However, rather than supporting a strict dichotomy between prediction and reaction, our results suggest that skilled interception behavior exists along a prediction-feedback continuum. In time-constrained interception scenarios, experts were found to generate earlier predictive saccades toward key target areas and to maintain more stable fixations at the predicted point of contact than novices. This strategic anticipatory positioning, coupled with efficient processing of online information, enabled skilled performers to execute more refined and efficient spatiotemporal coordination between visual input and motor actions.

The experimental manipulation involving the occlusion of late-phase visual information further highlighted distinct perception-action coupling strategies based on expertise. Unlike novices, skilled performers were more likely to flexibly integrate predictive eye movements with available online cues to cope with the sudden loss of sensory information, maintaining control of their motor responses within the functional limits of their sensorimotor system. Specifically, although no significant group differences emerged in the onset timing of predictive saccades across visibility conditions, skilled participants demonstrated a strategic adaptation in their spatiotemporal coordination under hidden conditions. This is evidenced by a significant increase in their eye-hand spatial error when the stimulus was occluded. This indicates that the flexible use of online information played a crucial role in their ability to regulate actions and sustain effective performance, even when their primary strategy was disrupted.

Despite its contributions, this study is not without methodological limitations. First, our experimental design, which used a controlled, straight-line trajectory, manipulated the availability of visual information rather than the inherent unpredictability of the stimulus's path. While participants were informed that the target's arrival location could vary, no actual path manipulation occurred. This distinction between reduced visual information and true path uncertainty should be explicitly considered when interpreting our findings (Márquez and Treviño, 2024a). Second, while our study provided spatiotemporal error metrics for the relationship between the eye, stimulus, and hand, we did not provide kinematic data on the hand's movement path. We hypothesize that these hand trajectory metrics would also differ by skill level (Márquez and Treviño, 2024b), and this is an important area for future research. Additionally, current eye-tracking technology presents challenges in accurately capturing subtle fixation movements during the latter stages of stimulus motion, limiting our ability to directly examine online links between visuomotor behavior and performance outcomes under stimulus-hidden vs. stimulus-unhidden conditions. Furthermore, data from only 20 participants (10 per group) were included in the final analysis due to participant withdrawal and the exclusion of anomalous eye movement data. This relatively small sample size may limit the generalizability of the findings and the statistical power to detect more nuanced effects.

Nevertheless, the findings offer meaningful insights into how long skilled performers can effectively process sensory information during interception tasks. Future research should further refine our understanding of perception—action coupling from the perspective of predictive and prospective control, particularly in relation to performers' levels of striking skill. This could be achieved by incorporating a broader range of motion trajectories, such as linear and curvilinear paths, and by systematically manipulating the timing of visual occlusion during stimulus motion. Such extensions would allow for a more comprehensive exploration of the underlying information-processing mechanisms and would contribute to a more nuanced understanding of how perceptual-motor coordination strategies vary with expertise.

## Data Availability

The raw data supporting the conclusions of this article will be made available by the authors, without undue reservation.
